# MiNEApy: enhancing enrichment network analysis in metabolic networks

**DOI:** 10.1093/bioinformatics/btaf077

**Published:** 2025-02-22

**Authors:** Vikash Pandey

**Affiliations:** Department of Molecular Biology, Umeå University, Umeå, 90187, Sweden

## Abstract

**Motivation:**

Modeling genome-scale metabolic networks (GEMs) helps understand metabolic fluxes in cells at a specific state under defined environmental conditions or perturbations. Elementary flux modes (EFMs) are powerful tools for simplifying complex metabolic networks into smaller, more manageable pathways. However, the enumeration of all EFMs, especially within GEMs, poses significant challenges due to computational complexity. Additionally, traditional EFM approaches often fail to capture essential aspects of metabolism, such as co-factor balancing and by-product generation. The previously developed Minimum Network Enrichment Analysis (MiNEA) method addresses these limitations by enumerating alternative minimal networks for given biomass building blocks and metabolic tasks. MiNEA facilitates a deeper understanding of metabolic task flexibility and context-specific metabolic routes by integrating condition-specific transcriptomics, proteomics, and metabolomics data. This approach offers significant improvements in the analysis of metabolic pathways, providing more comprehensive insights into cellular metabolism.

**Results:**

Here, I present MiNEApy, a Python package reimplementation of MiNEA, which computes minimal networks and performs enrichment analysis. I demonstrate the application of MiNEApy on both a small-scale and a genome-scale model of the bacterium *Escherichia coli*, showcasing its ability to conduct minimal network enrichment analysis using minimal networks and context-specific data.

**Availability and implementation:**

MiNEApy can be accessed at: https://github.com/vpandey-om/mineapy

## 1 Introduction

Adaptations of a cell’s metabolism to changing environments, including shifting nutrient availabilities, toxic compounds, and other external and internal perturbations, are often critical for the cell’s survival. To understand the mechanisms driving cellular metabolic adaptation and the processes that form the cell’s adapted metabolic network, context-specific metabolic models are invaluable tools. Constructing a context-specific genome-scale metabolic model involves integrating various types of omics data, such as gene expression, proteomics, metabolomics, or other relevant datasets, with a constraint-based genome-scale metabolic model.

Flux balance analysis (FBA) is a constraint-based approach applied to genome-scale metabolic networks (GEMs) and has become a widely adopted method for studying cellular metabolism (Orth *et al.* 2010b). The COBRA toolbox (Heirendt *et al.* 2019) and COBRApy (Ebrahim *et al.* 2013) are the most commonly used packages for conducting FBA-based studies. However, these studies often result in flux solutions that contradict bioenergetics due to the absence of thermodynamic constraints (Soh and Hatzimanikatis 2010). To address this issue, the pyTFA package was recently developed that integrates thermodynamic constraints into GEMs, effectively eliminating thermodynamically infeasible flux solutions (Salvy *et al.* 2019).

Genome-scale transcriptomics experiments have become a routine tool in biomedical research for measuring the mRNA expression profiles of thousands of genes. The major challenge is interpreting long lists of genes to gain insights into biological mechanisms. One approach to addressing this problem is gene set enrichment analysis, which divides a long list of genes into a smaller list of more interpretable pathways or groups of genes that share common biological functions (Subramanian *et al.* 2005, Kamburov *et al.* 2011a, 2011b, Väremo *et al.* 2013, Reimand *et al.* 2019). While these approaches can successfully provide biological insights, they use limited knowledge of intricate metabolic pathways and cannot capture complex metabolic states, such as flux routing, cofactor balancing, and byproduct generation in GEMs. To address this, a more general pathway concept based on elementary flux modes (EFMs) has been developed. EFMs are defined as minimal sets of irreversible reactions that operate at a steady state (Schuster and Hilgetag 1994, de Figueiredo *et al.* 2009). Additionally, context-specific EFMs can be identified using GEM and context-specific gene expression data (Rezola *et al.* 2013). However, enumerating all EFMs in a GEM can be computationally intractable; thus obtaining EFMs for a specific target metabolite synthesis requires adjustments in methods for computing EFMs.

To address this issue, a method called minimum network enrichment analysis (MiNEA) has been developed for identifying context-specific metabolic networks for a given list of metabolic tasks (Pandey and Hatzimanikatis 2019c). MiNEA computes alternative minimal networks (MiNs) for the biosynthesis of biomass building blocks (BBBs; Smith 1992) or metabolites characteristic of a cellular state, such as oxygen radicals. It then ranks MiNs using context-specific gene expression data. These features make MiNEA a versatile tool for exploring metabolic phenotypes and studying context-specific metabolism.

The proposed tool in this study, MiNEApy, is a Python-based framework for minimal network enrichment analysis, designed to enhance accessibility and versatility. MiNEApy builds upon the MATLAB code from the Pandey and Hatzimanikatis’ (2019) study, leveraging Python’s open-source nature and widespread popularity. While the MATLAB code utilized relative data for task enrichment, MiNEApy extends its functionality by incorporating absolute gene expression data, such as RPKM or FPKM values. This enhancement enables researchers to perform task enrichment analyses across diverse datasets and biological contexts.

## 2 Integrative methods for constraint-based modeling

In context-specific metabolic modeling methods like INIT (Agren *et al.* 2012) and TEX-FBA (Pandey *et al.* 2019) integrate gene or protein expression data into metabolic models, where TEX-FBA incorporates thermodynamic constraints. Tools like INTEGRATE (Di Filippo *et al.* 2022) and REMI (Pandey *et al.* 2019a) extend capabilities by including metabolomics data. These methods are effective for exploring and comparing whole metabolic states across different conditions, enabling the identification of differentially regulated reactions. However, further analysis, such as subsystem enrichment, is often required to interpret these differences.

Another tool, scFEA (Alghamdi *et al.* 2021), estimates flux from single-cell RNA-seq data using a factor graph and neural network approach. Although it does not account for mass balance or thermodynamic constraints, it is effective in identifying metabolic modules within specific cell types.

Our proposed tool, MiNEApy, serves as a valuable tool for downstream analysis in methods such as INIT, REMI and INTEGRATE. After these methods identify differentially regulated reactions, MiNEApy helps determine which metabolic tasks are enriched, providing deeper insights into context-specific metabolic sub-networks. MiNEApy complements tools like scFEA by performing enrichment analysis after the identification of differential cell markers for a specific cell type. Additionally, MiNEApy prioritizes ranking subnetworks based on experimental data and predefined metabolic tasks, rather than systematically incorporating constraints from experimental data. This makes it particularly useful for investigating task-specific networks rather than broad metabolic states.

## 3 Implementation and features

**Figure 1.**
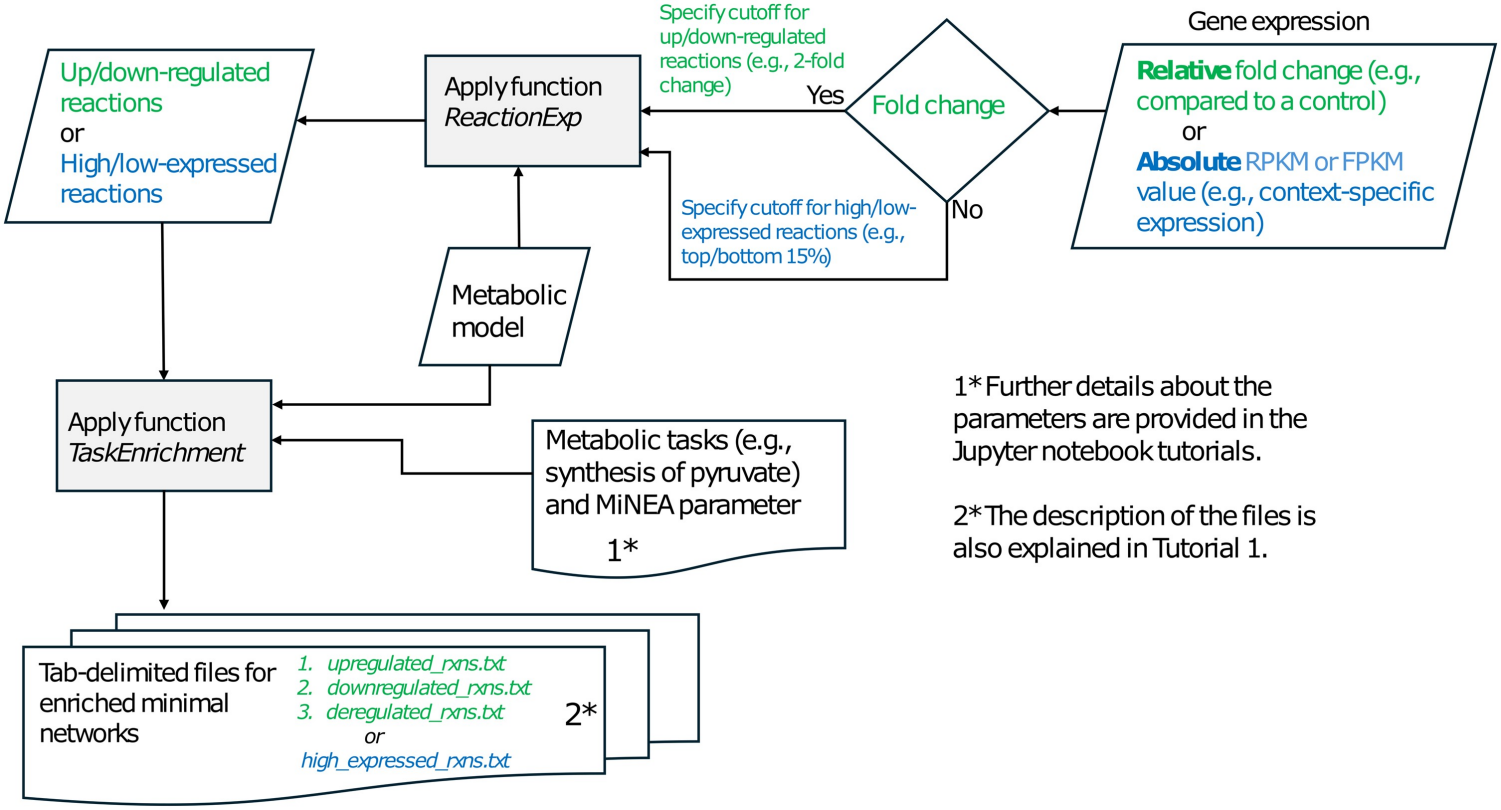
Illustration of the MiNEApy workflow. The MiNEApy workflow integrates a metabolic model, metabolic tasks, and gene expression data (such as fold change, RPKM, or FPKM values). The function “ReactionExp” processes the metabolic model along with either fold change data for identifying up- and down-regulated reactions, or RPKM/FPKM data for condition- or tissue-specific analysis. The output from “ReactionExp” is then used as input for the “TaskEnrichment” function, which also takes the metabolic model and MiNEApy parameters (including a list of metabolic tasks and method-specific parameters). The “TaskEnrichment” function generates tab-delimited files with ranked alternative minimal metabolic networks based on *P*-values, providing enriched networks for the given input conditions.

In Fig. 1, the summary of MiNEApy’s workflow is illustrated. Inputs for minimum network enrichment analysis (MiNEApy) are: (i) a constraint-based metabolic model, (ii) a list of metabolites that can be synthesized through the model, and (iii) gene expression data (Pandey and Hatzimanikatis 2019). MiNEApy generates output in the form of tab-delimited files containing *P*-values, which rank alternative metabolic networks based on the enrichment of gene expression data. MiNEApy supports models with and without thermodynamic constraints. The pyTFA package can be used to incorporate thermodynamic constraints into the model (Salvy *et al.* 2019). Once the model format is finalized, MiNEApy computes flux variability analysis to identify blocked reactions that do not carry flux. The MiNEApy formulation ignores variables related to the blocked reactions, thereby speeding up the optimization process.

MiNEApy identifies feasible minimum-size networks (MiNs) that are active for specific metabolite synthesis or for the synthesis of BBBs. The calculation of MiNs relies on various parameters, including alternative MiNs, diverged MiNs, and solvers (refer to tutorials). These parameters can be modified to determine the number of alternative and diverged MiNs, with the diverge parameter quantifying the difference between two alternative MiNs based on the number of distinct reactions. The enumeration of MiNs involves solving a mixed-integer linear programming problem within the MiNEApy framework, which can be computationally demanding. To address this, MiNEApy employs a time limit parameter to terminate problem solving after a user-defined duration.

A multivariate hypergeometric test, first introduced by Rezola *et al.* (2013), considers both highly and lowly expressed reactions within the same statistical framework. This method identifies EFMs that encompass a high number of up-regulated reactions while minimizing the inclusion of down-regulated reactions. Pandey and Hatzimanikatis (2019) further applied this test to identify significantly up-regulated or down-regulated MiNs in relative cases, or high-expressed MiNs in absolute cases. To obtain up-regulated MiNs, MiNEApy selects MiNs that contain an elevated number of up-regulated reactions and as few as possible down-regulated reactions; for identifying down-regulated MiNs, MiNEApy selects an increased number of down-regulated reactions and a decreased number of up-regulated ones. To identify highly expressed MiNs, MiNEApy selects a maximum possible number of highly expressed reactions and a minimum possible number of low-expressed reactions.

The output of MiNEApy is a tab-delimited text file containing information about MiNs and enrichment measures, such as percentages and *P*-values. This output allows users to identify which MiNs of certain metabolic tasks are enriched in a given context. These MiNs can then aid in exploring biochemical transformations and understanding context-specific metabolism.

The Python package MiNEApy is designed to integrate with COBRApy (Ebrahim *et al.* 2013) and pyTFA (Salvy *et al.* 2019), and utilizes Optlang (Jensen *et al.* 2017) as a modeling language for solving mathematical optimization problems.

MiNEApy includes three tutorials that demonstrate the enumeration of minimal networks and the integration of gene expression data. The first two tutorials focus on *Escherichia coli*: one uses a reduced core metabolic model (Orth *et al.* 2010a, Schellenberger *et al.* 2010), and the other utilizes the genome-scale model iJO1366 (Orth *et al.* 2011). For the enrichment analysis in both *E. coli* tutorials, we utilize gene expression data from the GEO database (Barrett *et al.* 2013), specifically the dataset GSE152445, which captures the transcriptional response of *E. coli* to a high dose of ciprofloxacin (Bie *et al.* 2023).

The third tutorial explores a genome-scale model of the *Plasmodium berghei* parasite (Stanway *et al.* 2019), using gene expression data from its exo-erythrocytic stages (Caldelari *et al.* 2019) to analyze liver-stage-specific metabolic pathways. The results from all three tutorials are compiled in Table S1, available as supplementary data at *Bioinformatics* online.

## 4. Conclusions

I developed the MiNEApy software package to perform minimal network enrichment analysis using constraint-based models and context-specific gene expression data. This package can be used to understand context-specific metabolism across different organisms by analyzing gene expression data measured under various conditions or perturbations. It offers a range of analyses valuable to the biomedical research, systems biology, and metabolic engineering communities. Our Python package is available on GitHub at https://github.com/vpandey-om/mineapy and has been archived on Zenodo at https://zenodo.org/records/14784442.

## Supplementary Material

btaf077_Supplementary_Data
